# Acute immune responses in zebrafish and evasive behavior of a parasite – who is winning?

**DOI:** 10.3389/fcimb.2023.1190931

**Published:** 2023-07-05

**Authors:** Heidi Mathiessen, Sebastian Kjeldgaard-Nintemann, Carlota Marola Fernandez Gonzalez, Cyril Henard, Julie Algreen Reimer, Sara Vebæk Gelskov, Moonika Haahr Marana, Louise von Gersdorff Jørgensen

**Affiliations:** ^1^ Experimental Fish Models (ExFiMo), Department of Veterinary and Animal Sciences, Faculty of Health and Medical Sciences, University of Copenhagen, Frederiksberg, Denmark; ^2^ Department of Plant and Environmental Sciences, Faculty of Science, University of Copenhagen, Frederiksberg, Denmark; ^3^ Department of Science and Environment, Roskilde University, Roskilde, Denmark

**Keywords:** zebrafish, innate immunity, imaging, inflammation, neutrophils, macrophages, *Ichthyophthirius multifiliis*

## Abstract

The protozoan parasite *Ichthyophthirius multifiliis* is an economically important parasite for the aquaculture- and ornamental fish industry. The parasite is abundant worldwide and infects the skin, gills and fins of freshwater fish species. For approximately the last fifty years the innate and protective immune mechanisms induced by *I. multifiliis* have been in focus in different fish hosts. By utilizing transgenic zebrafish, new tools to investigate this have emerged. The aim of this study was therefore to elucidate early immune responses in zebrafish larvae by using gene expression and *in vivo* imaging of neutrophil and macrophage behavior during infection. For the first time, zebrafish larvae were infected with the parasite and infection dynamics, parasite size and host-parasite interactions were investigated. Results showed that the larvae responded with mild inflammation and that the 12 compared to 5 days post fertilization larvae were significantly less susceptible. It was furthermore observed that neutrophils and macrophages were attracted to the parasites and that neutrophils reacted with neutrophil extracellular traps (NETs) when fighting the parasite. The parasite was rotating vigorously, presumably to impede the neutrophils and macrophages from attaching to it but on rare occasions, neutrophils and macrophages were able to kill the parasite. Based on these observations, we concluded that the parasite uses the rotation as an immune evasive strategy and that the zebrafish larvae respond with high activity from neutrophils and macrophages locally but systemically only with mild inflammation.

## Introduction

*Ichthyophthirius multifiliis* is a protozoan ciliated parasite that infects almost all freshwater fish species worldwide and causes white spot disease with high morbidity and mortality (Jørgensen, 2017). The disease is a major problem both for the aquaculture- and the ornamental fish industry with enclosed systems that contain a high density of fish as it causes severe economic losses for the farmers and significant suffering for the fish (Dickerson and Findly, 2014). Thus, research within immunological responses of the host and parasite behavior is a relevant field in relation to development of prophylactic measures and safe control methodologies. In nature, the parasite and the fish are co-existing. Fish density is much lower compared to fish production systems and white spot disease is a frequent condition with low intensity not harming the fish significantly. Whether fish experience a high or low level of infection, an intriguing host-parasite relationship is taking place and deserves attention.

The parasite has four life stages consisting of the infectious free-swimming theronts, the parasitizing trophonts, the free-swimming tomonts and the pre-theronts called tomites. Within 24 h at 15°C, the theronts must find a suitable host and as soon as the theronts penetrate the skin, fins and gills of the host fish they will settle above the basal lamina and be covered by at least one cell layer of host tissue (Ewing et al., 1985; Ventura and Paperna, 1985). Subsequently, they transform into trophonts (the transformation stage between theront and trophont is, in this paper, termed early trophont) and start feeding on host materials after some hours (Matthews, 2005). This parasitizing stage is, as the rest of the life cycle, dependent on temperature and will last longer at lower temperatures (Aihua and Buchmann, 2001). When the trophont is mature, it will exit the host and become a free-swimming tomont. The tomont will settle on bottom substrates and become encysted in gelatinous material (Matthews, 2005). Within this so-called tomocyst, tomites will develop by binary fission and following a maturation period, become theronts that will exit the cyst and search for new hosts (Matthews, 2005). One tomocyst can produce between 50-1000 new theronts and the infection pressure can quickly become very intense (Hines and Spira, 1974; Dickerson and Clark, 1998; Matthews, 2005; Jørgensen, 2017).

Zebrafish have become a very popular model species for a number of reasons, previously described in numerous articles and reviews (Dooley and Zon, 2000; Traver et al., 2003; Lieschke and Currie, 2007; Sullivan and Kim, 2008; Shive, 2013; Brugman, 2016; Bradford et al., 2017; Cayuela et al., 2018; Saleem and Kannan, 2018; Carnovali et al., 2019; Bhagat et al., 2020; Jorgensen, 2020; Choi et al., 2021). Larvae only possess innate immune responses for the first four weeks of life, whereas adult zebrafish have both innate and adaptive immune mechanisms (Novoa et al., 2006). In previous studies, adult zebrafish have been shown to be more resistant towards *I. multifiliis* compared to most other fish species (Cherry, 2003; Jorgensen, 2016a), opening up an opportunity for investigating mechanisms of natural protection. To elucidate the innate responses in this natural protection, we chose to infect zebrafish larvae. The host-parasite relationship has until now only been studied in adult zebrafish where it was shown that two day old parasites are able to ingest and kill immune cells, which are approaching and attacking the parasite (Jorgensen, 2016b; Jorgensen et al., 2018). It was furthermore documented how trophonts continuously rotate and move around under the epidermis of the host fish (Jorgensen, 2016b).

The early naïve responses (responses from fish that encounter the pathogen for the first time) towards especially heavy infections with *I. multifiliis* in adult or juvenile fish include inflammatory responses (Ewing and Kocan, 1992; Cross, 1994; Gonzalez et al., 2007a; Jorgensen et al., 2018; Syahputra et al., 2019) and activation of the adaptive arm of the immune system (Dickerson and Findly, 2017; Jorgensen et al., 2018; Syahputra et al., 2019). It has been described how leucocytes get attracted to the parasite but are unable to harm it (Ventura and Paperna, 1985; Cross and Matthews, 1993; Cross, 1994). The parasite is, on the contrary, able to harm or neutralize the cells (Ewing et al., 1985; Ewing and Kocan, 1992; Jorgensen, 2016b). Leucocytes such as macrophages, neutrophils, basophils and eosinophils are known to be major players in the acute immune response (Ventura and Paperna, 1985; Cross and Matthews, 1993; Jorgensen, 2016b). Macrophages are important effector cells in inflammation, tissue repair and regeneration (Bohaud et al., 2021). They are phagocytic cells important for the host defense against pathogens but also function like a vacuum cleaner clearing up apoptotic cell debris during development and inflammation. They connect the innate and the adaptive arm of the immune system and regulate immune responses (Mosser and Edwards, 2008; Renshaw and Trede, 2012; Bohaud et al., 2021). Neutrophils are mostly found in tissues, not in the blood, and represent the majority of leucocytes in zebrafish larvae from two days of age (Deng et al., 2011; Renshaw and Trede, 2012). These cells are also phagocytic, kill pathogens and repair wounds (Renshaw et al., 2006). Both neutrophils and macrophages have pattern recognition receptors (PRRs) that recognize pathogen associated molecular patterns (PAMPs) and damage associated molecular patterns (DAMPs), originating from pathogens and tissue damage, respectively (Alvarez-Pellitero, 2008; Li et al., 2017). Using a fluorescent neutrophil reporter line Tg(MPX:eGFP)^i114^ it was demonstrated that neutrophils were actively involved in both the innate and adaptive response against the parasite in adult fish. In the same study, gene expression analyses revealed that a classical proinflammatory and a Th2-like response were induced in immunized zebrafish (Jorgensen et al., 2018). *I. multifiliis*-induced tissue damage and the pathogen itself will activate cells bearing PRRs, which may initiate inflammation and an acute phase response. This, however, does not resolve the disease in most fish species (Ewing et al., 1985; Cross, 1994; Jorgensen, 2016a; Jorgensen, 2016b; Jorgensen et al., 2018) even though many parasites are killed or die immediately upon entry (Ventura and Paperna, 1985). Infections with few parasites induce a very limited local response securing compatibility between the host and the parasite and both species will survive (Ventura and Paperna, 1985).

In this study, we have for the first time infected zebrafish larvae with *I. multifiliis* to elucidate the acute innate immune response and investigate the host-parasite relationship. Several experiments were conducted to analyze: 1) infection dynamics of the parasite in 5 and 12 days post fertilization (dpf) larvae; 2) size of the parasite in larvae (5 dpf) and juvenile fish (28 dpf); 3) the inflammatory profile of 5 and 12 dpf larvae; 4) behavior of neutrophils and macrophages in the response against *I. multifiliis* in 5 and 12 dpf larvae at 5 hours post infection (hpi); 5) real-time imaging of host-parasite relationships in 5 and 12 dpf larvae at 5 hpi.

## Materials and methods

To our knowledge, this is the first time zebrafish larvae have been experimentally infected with *I. multifiliis*. Therefore, some basic investigations were conducted, including size measurements of the growing parasite and estimation of infection levels. These findings complement the in-depth complex immunological and host-parasite studies. Furthermore, three different age groups of zebrafish (5, 12 and 28 dpf) were used to include the early-, middle- and late stage of innate immune responses. Not all investigations were conducted on all age groups due to limited availability of the parasites.

We followed all ethical considerations described in the associated license 2021-15-0201-00951 obtained from the Animal Experiments Inspectorate under the Danish Ministry of Environment and Food.

### Infection with *I. multifiliis*

Due to ethical considerations, it is not allowed to keep the *I. multifiliis* infection in the laboratory. Therefore we needed to obtain the parasite from a pet shop prior to each experiment and consequently used a different isolate with distinctive infection pattern in different experiments. Fish were euthanized with an overdose of the anesthetic tricaine methanesulfonate (MS222, Sigma-Aldrich). Subsequent steps were conducted at 26-27°C. Following euthanization, the fish were immediately transferred to sterile-filtered (Minisart®Syringe Filter, pore size 0.45 μm) facility water where the parasites exited the fish, settled on bottom surfaces and within the next 24 hours infective theronts appeared. The concentration of theronts was determined by counting 5 subsamples of 10 µL water and calculating the average theront density. The infection was conducted with a calculated amount of water with theronts to obtain the predetermined infection level, which was added directly to relevant wells.

### Fish

In the experiment 5, 12, and 28 dpf larvae were used of an AB wildtype strain and a double transgenic line obtained by breeding Tg(MPX : GFP)^i114^ (Renshaw et al., 2006) and Tg(Mpeg:mCherry-CAAX)^sh378/+^ (Ellett et al., 2011). Water conditions in the facility were: pH 7.5, conductivity approximately 800 µS, water temperature 27°C and 10% of the water was exchanged every day and replaced with de-ionized water running through an RO installation.

### Experimental design

The *I. multifiliis* infection lasted maximally 72 h and within this period all sampling was conducted. We chose to collect samples for qPCR and imaging within the first 8 hours of infection because we have previously observed that the larvae sometimes are able to expel the parasites within approximately 7.5 hours.

### Determining the infection level

To estimate the level of infection an experiment was set up as seen in **Supplementary Figure 1A**.

Four 6-well plates were used for the experiments. All 24 wells contained 10 WT zebrafish larvae in 5 mL sterile filtered facility water and the experiment was performed in triplicate. Each well was inoculated with a determined concentration of theronts and 24 h after infection all larvae were anaesthetized in 150 mg/L MS222, and the parasites, which successfully had infected the larvae, were counted. This setup was used both for 5 and 12 dpf larvae. Triplicate data was analyzed using a Kruskal-Wallis test in GraphPad Prism 9 (GraphPad Software, LLC) and if no significant difference was evident the data was pooled. The difference between the data for 5 and 12 dpf larvae was analyzed using a non-parametric Student’s t-test (p<0.05).

### Measuring the size of *I. multifiliis*

To estimate the increase in size of the parasite and correlating this to age of the fish we infected 5 dpf larvae with 50, 100 and 300 theronts/larva and 28 dpf zebrafish with 1000 theronts/fish. Images were obtained 5, 24, 48 and 72 h following infection. At 72 h almost all parasites had left the fish. Five-day old larvae were embedded as described in the Imaging section and euthanized following imaging. The 28 dpf fish were euthanized in an overdose of MS222 and bright field images were obtained as soon as the fish became immobile. The Zen lite software (Zeiss) was used to measure the size of the parasites. First the scalebar was implemented to the images and subsequently, a line drawing tool was used to precisely measure the diameter of the parasite. Data was analyzed using a linear regression test in GraphPad Prism 9.

### qPCR

To conduct qPCR for larvae at both 5 and 12 dpf in triplicate the experiment was set up as shown in **Supplementary Figure 1B**. We used four 6-well plates and placed 10 WT larvae in each of 18 wells in 5 mL sterile filtered facility water. Half of the wells contained uninfected larvae serving as time point controls and the other half contained infected larvae (50 theronts/larva). All 10 fish in each well were sampled at 2, 5 and 8 hpi. When sampling, MS222 was added to each well until larvae were anaesthetized. Subsequently, larvae were collected and placed in Eppendorf tubes. Here they were given an overdose of MS222 (500 mg/L) and after one minute the fluid was removed and 200 µL RNAlater (Sigma-Aldrich) was added. Tubes were kept at 4°C for 24 h and subsequently at -20°C until further processing. cDNA was generated and qPCR reactions conducted as described in Marana et al., 2022 (Marana et al., 2022). The panel of genes investigated for this experiment can be found in **Supplementary Table 1**. An average of three housekeeping genes (*β-actin, elf-α* and *rpl13*) was used. Fold change was calculated according to Livak and Schmittgen (2001). Data was analyzed using a Student’s t-test assuming a Gaussian distribution, comparing each age group to the time point control.

### Imaging

For imaging, a standard procedure for sample preparation and embedding was used. Briefly, 5 and 12 dpf larvae were anaesthetized in 150 mg/L MS222 5 hpi and subsequently transferred to dishes (WillCo-dish) with thin glass bottoms suited for microscopy. Here the water and MS222 was removed, and larvae were embedded in 40°C low melting point agarose as follows: a heated low melting point agarose (Sigmal Aldrich) was added to the anaesthetized larvae. The low melting point gel consists of 89.2% sterile filtered fish facility water, 10% 1.75g/L MS222 and 0.8% low melting point agarose (Sigma-Aldrich). The larvae in the gel were aligned to the bottom of the glass bottomed petri dish. The gel with larvae was allowed to solidify for 20 minutes and was subsequently covered in sterile filtered facility water containing 150 mg/L MS222 to keep the larvae anaesthetized. Afterwards, images were obtained either by a stereo fluorescence microscope (Zeiss V8) or by confocal laser scanning microscopy (Leica Stellaris 8, Leica Microsystems). For both microscopes, settings detecting green fluorescent protein (GFP) (excitation and emission peaks at 488 nm and 510 nm) and mCherry (excitation and emission peaks at 587 nm and 610 nm) as well as for the acquisition of bright field images were used.

Images from the Zeiss microscopes were imported into Fiji Image J and adjusted to optimize visualization (brightness/contrast) of neutrophils, macrophages, and parasites. Confocal images and videos were recorded with a HC PL APO CS2 40x/1.25 glycerol immersion objective and laser lines at 489 nm and 587 nm and detection settings 494 nm to 572 nm and 593 nm to 839 nm for GFP and mCherry detection, respectively. Videos were recorded as Z-stacks (30 µm depth, 25 steps) and time series (4 stacks per minute). The presented video represents a maximum intensity projection of the three-dimensional data. In the fluorescence channels, the median noise reduction algorithm (radius 2, 2 iterations) of the imaging software (Leica Application Suite X, Leica Microsystems) was applied.

In order to observe host-parasite interactions, images and videos were captured to illustrate parasite behavior and immune cell responses (i.e. neutrophils and macrophages). Four different scenarios that were encountered at least twice are described in the results section. Some events are rare, and their frequency cannot be estimated with the limited data acquired in this study.

### Counting phagocytes

To estimate the neutrophil and macrophage population in 5 and 12 dpf zebrafish larvae, phagocytes were counted using images from a stereo fluorescence microscope (Zeiss V8). The anaesthetized larvae were embedded as described in the Imaging section. For the neutrophil and macrophage prevalence analysis, images of the tail region of un-infected fish were captured and the total number of phagocytes in the 5 and 12 dpf larvae was manually counted between the anal opening and the caudal fin using the multi point function in Fiji Image J (**Supplementary Figure 2**). The difference between the data for 5 and 12 dpf larvae was analyzed using a Students t-test (p<0.05).

## Results

Five and 12 day old zebrafish larvae were infected with *I. multifiliis* to elucidate infection dynamics as well as the host immune response and the host-parasite relationship. Furthermore, the size of the parasites was followed in 5 and 28 day old fish during 72 hours of infection.

### Infection dynamics

**Figure 1:**
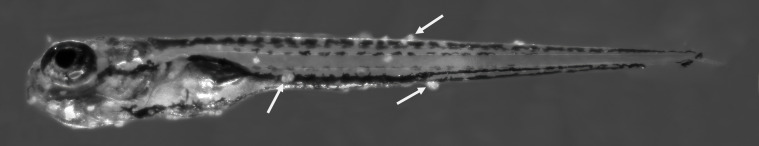
Five day old zebrafish larva (5 mm) infected with *I. multifiliis* parasites. The image was obtained 24 h after infection. White arrows point to three out of many trophonts in the skin.

**Figure 2:**
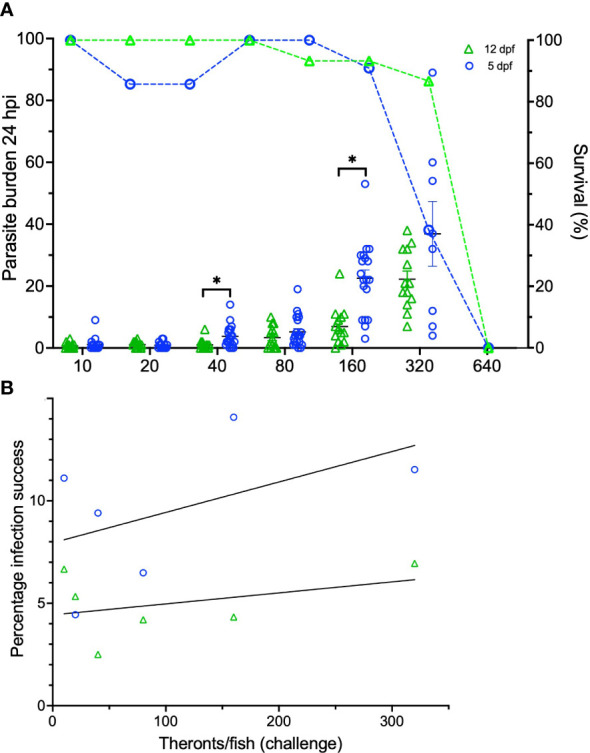
Infection data on zebrafish larvae 5 and 12 dpf infected with *I. multifiliis*. **(A)** The parasite burden is shown in relation to infection pressure. Survival curves (dotted lines) are associated to the right Y-axis. **(B)** Percentage of infection success is shown in relation to infection pressure. *indicates statistical difference with P<0.05 using a Student’s t-test.

For the first time, zebrafish larvae were experimentally infected with *I. multifiliis* (**Figures 1**, **2**). Because of the small size of the larvae and the relatively large size of the parasite, it was possible to count the total amount of parasites on the two different age groups of zebrafish larvae (**Figure 2A**). There was no significant difference between the triplicate groups, and they were therefore pooled. Five dpf larvae carried significantly higher parasite burdens compared to 12 dpf larvae at infection pressures of 40 and 160 theronts/larva at 24 hpi. All larvae exposed to higher concentrations than 320 theronts/larva became moribund and were euthanized. The percentage of parasites that successfully established in the skin or the fins of the fish (**Figure 2B**) showed a low level of correlation to infection pressure (for 5 dpf larvae R^2 = ^0.25 and for 12 dpf larvae R^2 = ^0.14). Approximately 10% and 5% of the theronts successfully established in the 5 and 12 dpf larvae, respectively.

### Duration of infection and size of the parasite

**Figure 3:**
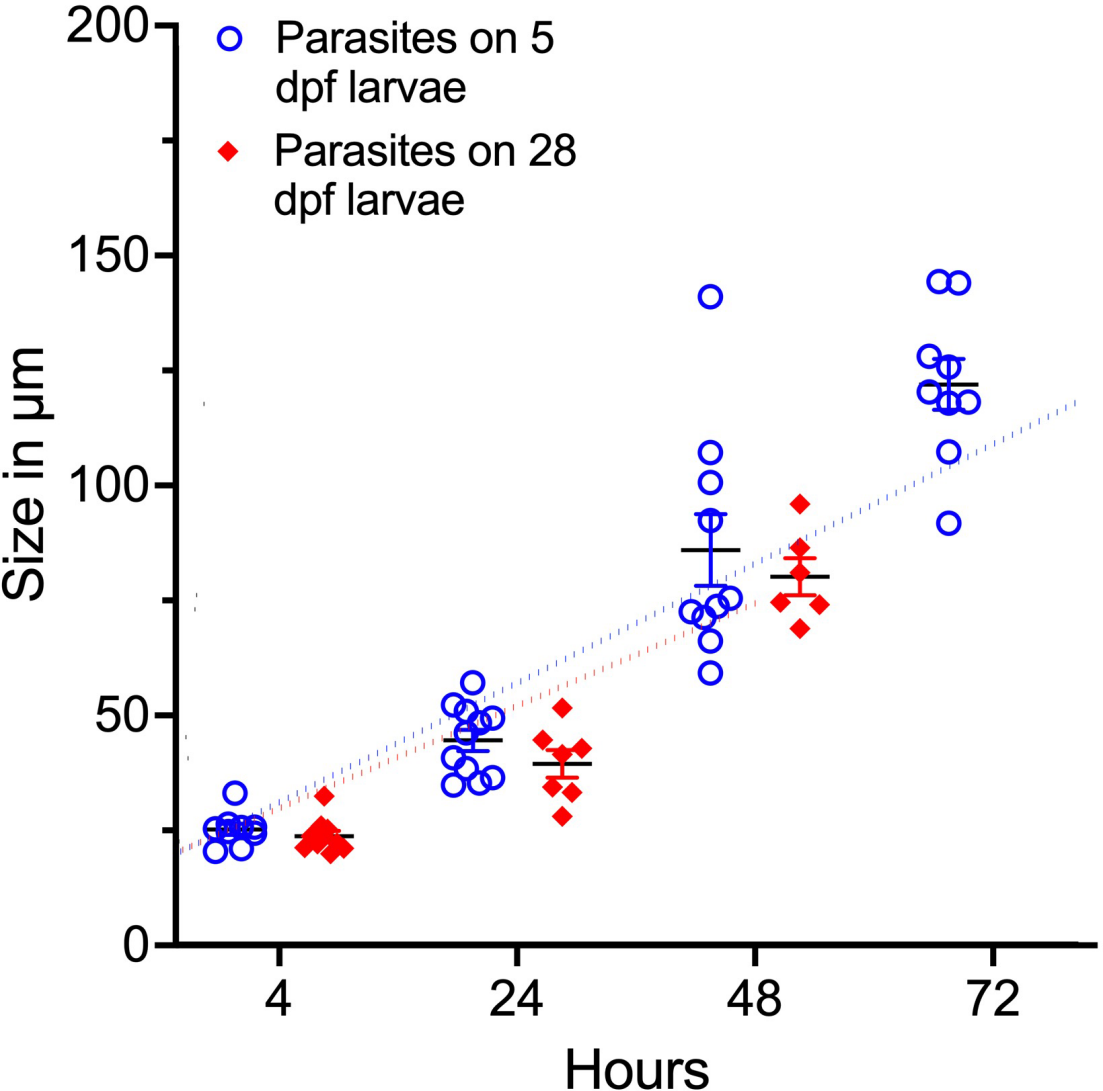
Parasite size (diameter) related to time of infection. Dotted lines represent linear regression. There is a positive correlation between size and period of infection (r^2^=0.85 for 5 dpf and r^2^=0.89 for 28 dpf).

In the 28 dpf fish, no parasites were found at 72 hpi and at 96 hpi the parasites that were left on the 5 dpf larvae exited the fish during the handing process before images could be obtained. The *I. multifiliis* diameter was measured to estimate the growth of the parasite on zebrafish larvae (**Figure 3**). There is a positive correlation between the size of the parasite and the time from infection (r^2 = ^0.85 for 5 dpf and r^2 = ^0.89 for 28 dpf). Furthermore, there is no significant difference between the size of the parasites on 5 and 28 dpf larvae.

### Gene expression

**Figure 4:**
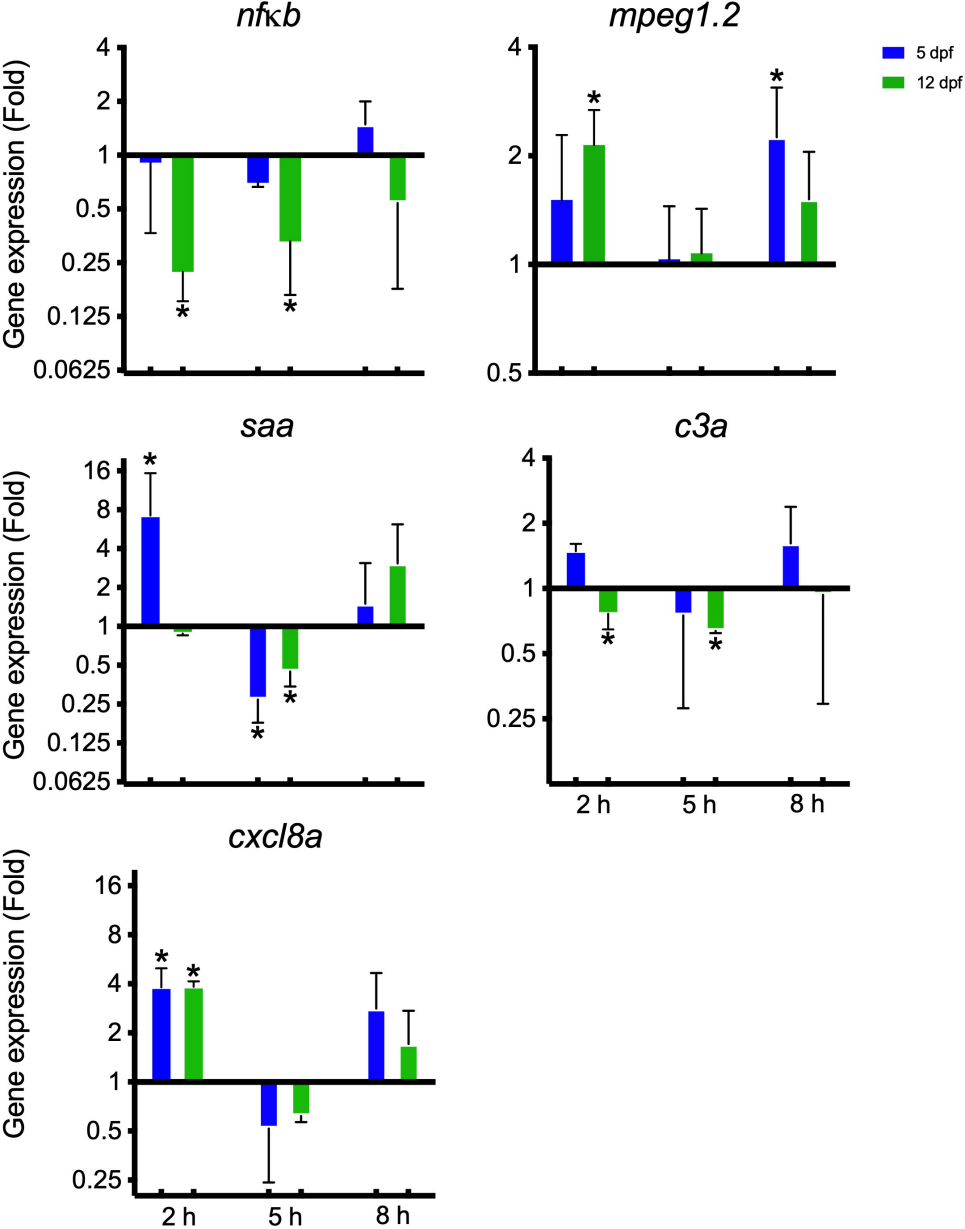
Gene expression conducted on zebrafish larvae 5 and 12 dpf infected with *I. multifiliis*. The figure shows significantly regulated genes out of a whole panel of immune-relevant genes investigated (**Supplementary Table 1**). * indicates statistical significance with P<0.05.

A panel of immune-relevant gene expressions were analyzed using qPCR. Most genes were not significantly regulated except for five (**Figure 4**). At two hpi, the gene encoding serum amyloid A (*saa*) was upregulated in the 5 dpf group; the macrophage-expressed gene (*mpeg1.2*) was upregulated in the 12 dpf group; the C-X-C motif ligand 8 (*cxcl8a*) was upregulated for both 5 and 12 dpf larvae whereas *nfκb* and *c3* were downregulated for the 12 dpf larvae. At 5 hpi, the genes for nuclear factor kappa B (*nfκb*), the complement factor 3 (*c3a*) and *saa* were downregulated in the 12 dpf group. In the 5 dpf larvae *saa* was also downregulated at 5 hpi. At 8 hpi, *mpeg1.2* was upregulated in the 5 dpf group.

### Imaging

To investigate the early role of neutrophils and macrophages in the host response to the parasite and to investigate the parasite behavior, larvae of a double transgenic reporter line with fluorescent neutrophils (green) and macrophages (red) were infected with *I. multifiliis*. Images and videos were obtained with live fish and live parasites at 5 hpi.

**Figure 5:**
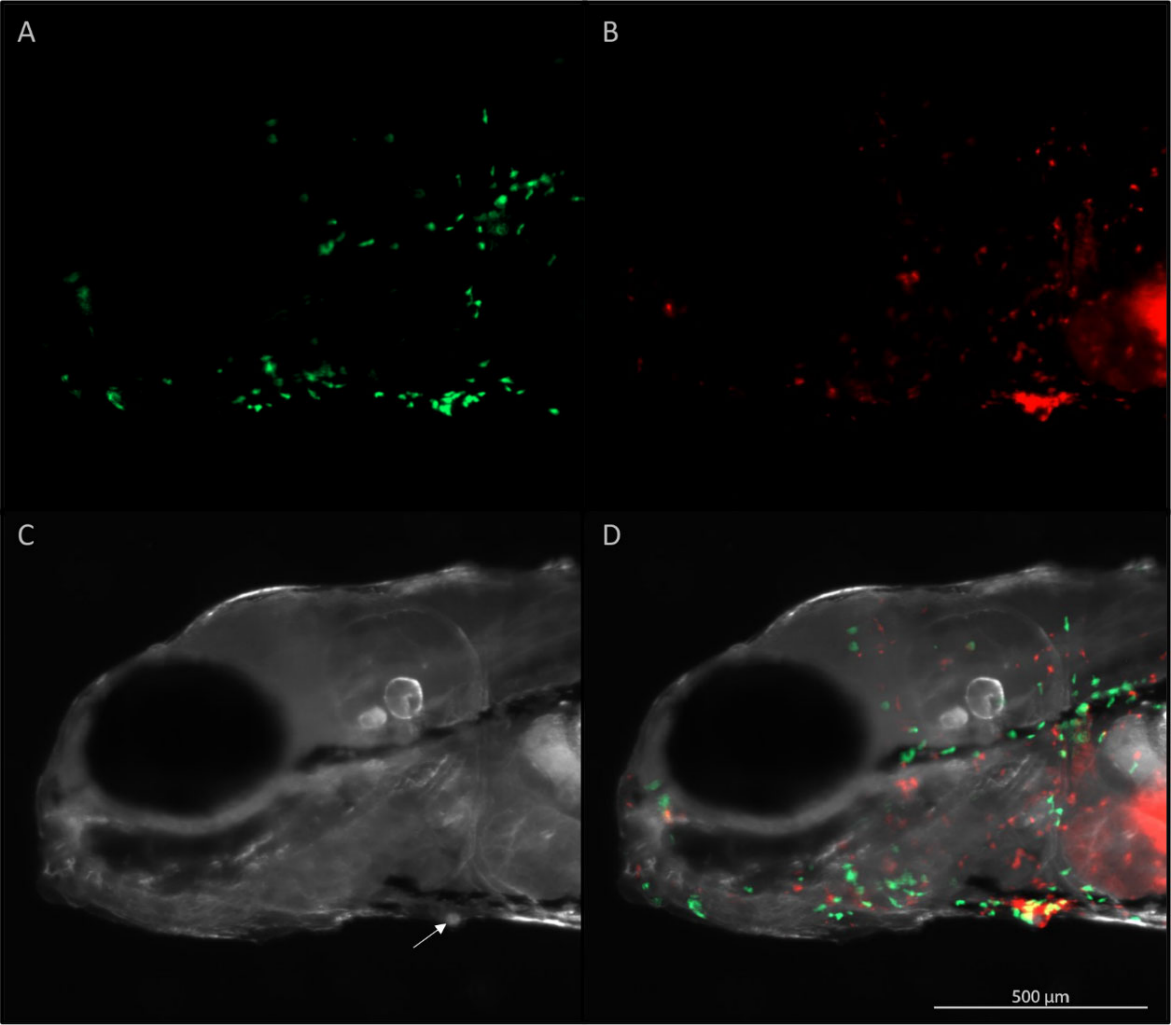
Live imaging of the head of a five-day old zebrafish five hours after infection with *I. multifiliis*. A parasite is found on the ventral side (arrow) of the fish. **(A)** Green fluorescent neutrophils, **(B)** red fluorescent macrophages, **(C)** white light illumination, **(D)** A merge of **(A–C)**. Videorecording of this parasite rotating is found in **Supplementary Video 2**.

**Figure 6:**
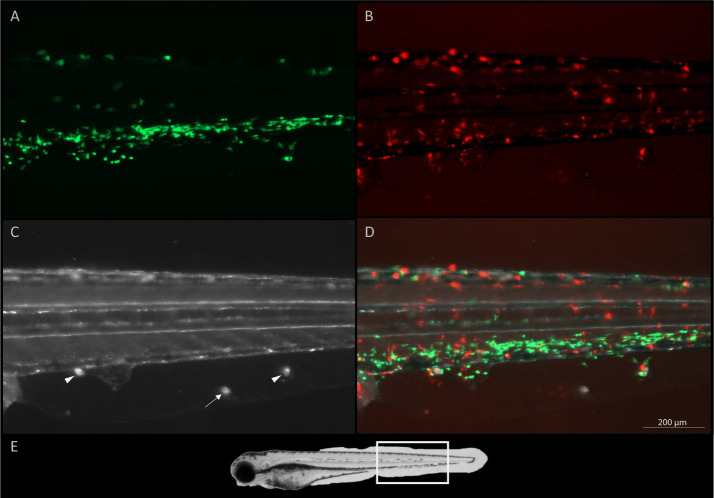
A twelve-day old zebrafish larva, five hours after infection with *I. multifiliis*. Three parasites are found on the ventral side (arrows and arrow heads) between the anal opening and the tail fin of the fish. **(A)** Green fluorescent neutrophils, **(B)** red fluorescent macrophages, **(C)** white light illumination, **(D)** A merge of **(A–C, E)** overview of image area marked with a white rectangle on a whole larva. A videorecording of these parasites rotating is found in **Supplementary Video 3**. Parasites surrounded by phagocytes are shown with arrow heads and a parasite appearing undetected by the immune system is indicated by an arrow.

Four different host-parasite interactions were observed (but not quantified) during imaging of 5 and 12 dpf infected larvae, and these data are used to support interpretation of the infection dynamics, the phagocyte count and the gene expression data:

1) Phagocytes surrounded the parasites but had limited effect (**Figure 5**). In at least 5 images of different parasites this scenario was observed and most of the time both neutrophils and macrophages were near the parasite but sometimes only one of the cell types was present.
2) The parasites were able to go unnoticed by the phagocytes (**Figure 6**). This scenario was observed on at least 5 images of different parasites and often unnoticed parasites were located in the fin.
3) Phagocytes were able to kill the parasites (**Supplementary Video 1**). This situation was observed at least twice but only videorecorded once.
4) Parasites left the interstitial space especially when surrounded by many phagocytes. This was observed at least three times. It was however, also observed that parasites sometimes left the interstitial space without being surrounded by phagocytes.

The rim of the interstitial space hosting the parasite was often covered by neutrophils and macrophages (**Figure 5**), but the parasite was at the same time still rotating and active (**Supplementary Video 2**). This particular parasite was followed for 8 h and the situation never changed.

In some cases when multiple parasites had infected the larvae (**Figure 6**), only some parasites were attracting phagocytes. The parasites were alive when images were obtained (**Supplementary Video 3**).

Using confocal microscopy, high resolution video recordings were captured illustrating phagocyte cell behavior when these cells were interacting with the parasites (**Supplementary Video 1**). This particular video shows how neutrophils and macrophages attack one parasite. During the sequence a neutrophil dies and green fluorescence from the dead cell is distributed in the interstitial space. Neutrophil extracellular trap (NET) formation was furthermore visible by balloon like structures (indicated in the video) for one neutrophil. An overview image of the parasite location in the larva is provided in **Supplementary Figure 3**.

### Phagocyte count

To estimate if 12 dpf zebrafish larvae had a larger population of phagocytes, the cells were counted in 5 and 12 dpf larvae. Even though the 12 dpf larvae had a higher mean number of both neutrophils and macrophages, no significant difference between the phagocyte numbers was evident (**Supplementary Figure 4**).

## Discussion

For the first time, zebrafish larvae were experimentally infected with *I. multifiliis* (**Figure 1**). Apart from the availability of various transgenic lines that enable immunological investigations, the use of zebrafish larvae is also of great value because of the species’ relative resistance to *I. multifiliis* infection. We conducted basic investigations on infection success and growth of the parasite in very young fish to evaluate the suitability of the zebrafish larvae model to study the host-parasite relationship. Using larvae younger than four weeks furthermore facilitated investigations of host innate response since the adaptive arm of the immune system only becomes functional from 4-6 weeks (Novoa et al., 2006).

### Infection dynamics

Five and 12 dpf larvae were able to receive an infection with *I. multifiliis* of up to approximately 320 theronts/larva without becoming moribund (**Figure 2**). Therefore, infection establishment in these two different age groups was investigated. Because a difference in infection success between the two age groups was evident (**Figure 2A**), the level of infection was not only a question of the infectivity performance of the theronts, but also a question of how severely the host responds to infection. The percentage of successful establishments of the parasites in the host’s skin (**Figure 2B**) did not change with a rising infection pressure (from 10-320 theronts/larva). The infection success is, therefore, only dependent on the compatibility between the host and parasite and not on short term stress possibly induced by infection pressure. The one-week older group of larvae (12 dpf) was more resistant towards the parasite (**Figure 2A**), indicating that the innate immune response or other factors at this life stage are more harmful for the parasite. Several infection trials were conducted for this investigation with varying infection success between the isolates. This led us to conclude that infection severity relies heavily on the parasite isolate of *I. multifiliis*. Virulence factors for this parasite still need to be described.

To investigate duration of the infection in zebrafish larvae and the increase of parasite size, the infection was conducted in 5 and 28 dpf larvae. The parasites stayed one to two days longer on the 5 dpf larvae demonstrating that the older fish created a more hostile environment around the parasite forcing it to exit prematurely. The size of the infective stage of the parasite is dependent on abiotic factors such as temperature and pH and with increasing temperatures and lower pH it is known that the theronts decrease in size (Aihua and Buchmann, 2001; Tange et al., 2020). In the zebrafish larvae, the trophonts (feeding stage) reached a much smaller size compared to what has been observed in other fish species (Matthews, 2005). It is possible that the size of trophonts is also affected by temperature, which may explain the relatively small size of these trophonts but more likely, the size is a result of the zebrafish larvae being a less compatible host for the parasite creating an unfavorable environment. The size of the parasites was similar across the two age groups implying that as long as the parasites are in the fish they are not impeded from eating.

During the parasitic stage the parasite is continuously growing and is ingesting a constantly increasing amount of fish material. It is known from a previous study that the parasite ingests active neutrophils (Jorgensen, 2016b) and neutralizes them but several studies have also demonstrated breakdown of leucocytes in the interstitial space (Ewing et al., 1985; Cross, 1994). In **Supplementary Video 1** a neutrophil death is real-time documented taking place in or around the interstitial space hosting the parasite. Whether or not the cell death is induced by the parasite remains to be demonstrated. It is also possible that the phagocyte’s oxidative defense mechanisms damage host’s own cells. Nonetheless, the response must be tightly regulated to avoid too much local self-damage.

### Gene expression

Gene expression analyses showed that the macrophage-expressed gene *mpeg1.2* specific for macrophages (Benard et al., 2015) was upregulated in the 5 and 12 dpf larvae (**Figure 4**). Additionally, imaging analyses documented activation of macrophages supporting at least a locally induced activation (**Figures 5**, **6**). Macrophages and neutrophils have a close collaboration and the two groups of cells chemically communicate (e.g. chemoattractants) to regulate the response (Bouchery and Harris, 2019). Mpeg1.2 has been shown to be induced by infection and functions in a pore-forming membrane complex associated with host defense (Benard et al., 2015). The 12 dpf larvae had not, however, a significantly higher numbers of neutrophils and macrophages when the populations were estimated (**Supplementary Figure 4**). The phagocytes may, on the other hand be more efficient at this stage. Before 5 dpf, only IRF8-dependent macrophages are produced but in larvae/juveniles older than 5-6 days, IRF8-independent macrophages are also developed from the kidney (Shiau et al., 2015). The latter cell type may be functionally different from IRF8 dependent cells (Qi et al., 2009) and perhaps represent a subset more effective in natural protection against *I. multifiliis* in older zebrafish. This is speculative and other factors may as well play a role, requiring further investigations. It is, however, known from mammals that different phenotypes of macrophages exist (Xue et al., 2014). In mammals it has been found that IRF8-silenced monocyte-derived macrophages display an M2 (healing) phenotype (Ototake et al., 2021) and that IRF8 inhibition negatively impacts M1 (inflammatory) macrophage mediators but not M2 mediators (Guo et al., 2017). It is also possible that since IRF8-independent macrophages are produced more in the head region compared to the tail region (Shiau et al., 2015), increase in the total number of macrophages was not detectable in the tail region. Phagocytes do, none the less, play an important role in protection against *I. multifiliis* in locally affected microenvironments, which was documented with images and videos (**Figures 5**, **6**; **Supplementary Video 1**).

Differentially expressed genes revealed a very early response including an upregulation of the gene encoding the proinflammatory chemokine Cxcl8, which is known to recruit neutrophils to the affected site through chemotactic stimuli (van der Aa et al., 2010; van der Vaart et al., 2012). Imaging supported that the larvae are responding with neutrophil activation in infected microenvironments (**Figures 5**, **6**; **Supplementary Video 1**) and in a few cases, neutrophils and macrophages managed to kill single parasites (**Supplementary Video 1**). Both PAMPs from the parasite and DAMPs from the infection site activate the immune cells. It has been described that NETs are utilized during neutrophil swarming in zebrafish, but whether the observed clusters around the parasites are swarms, remains to be determined (Isles et al., 2021). In this study, for the first time, NETs as a response to *I. multifiliis* early infection has been documented.

The apolipoprotein SAA, which is involved in the acute phase response and more specifically involved in the inflammatory process and acting as chemoattractant (Gonzalez et al.,; Kania et al., 2014), has been shown to be highly upregulated in both carp, rainbow trout and zebrafish infected with *I. multifiliis* (Gonzalez et al.,; Jorgensen et al., 2008; Jorgensen et al., 2018). In this study, a minor upregulation of *saa* in the 5 dpf larvae, immediately after infection, may indicate a role for SAA in the acute response towards infection. The gene is, however, downregulated at 5 hpi for both 5 and 12 dpf larvae indicating that the contribution of this acute phase protein is transient.

Nfκb plays an important part in the development (Correa et al., 2004) and in the regulation of inflammatory and immune responses (Saleh et al., 2019). It is activated by the proinflammatory cytokines Il1β and Tnfα and is also associated with the innate immune cell inflammatory response (Nivon et al., 2012; Shih et al., 2015). The downregulation observed here could indicate restriction of the immune and inflammatory responses to limit self-damage to the host. With a limited response during a mild infection the host and parasite become compatible and the parasite can co-exist in the fish host (Ventura and Paperna, 1985). Zhao et al., 2013 on the other hand found upregulation of TAK1 from 6 and 12 h in spleen and skin respectively in 50 g grass carp infected with *I. multifiliis*, which downstream activates the nfκb pathway (Zhao et al., 2013). Our sampling could be too early to detect an activation of the nfκb pathway or as, mentioned by Ventura and Paperna 1985: the carp species is, compared to other fish species infected with *I. multifiliis*, the only one reacting with an intense cellular infiltration of the epithelial layer during the early stage of infection (Ventura and Paperna, 1985).

A general trend was, that the larvae reacted with mild proinflammatory responses at 2 and 8 hpi and for 4 out of 5 genes a neutral- or downregulation at 5 hpi. We suggest that the 2 hpi response is due to mechanical damage, attempts to penetrate and penetration of the epithelium by the parasites. At 5 hpi most parasites that were able to enter the fish may have settled in the interstitial space and may immunosuppress immune responses. At 8 hpi the larvae may react to some of the early trophonts. Many genes did not significantly change expression pattern and in this setup, it is possible that the local responses at infection sites drowned in the global expression.

### The host-parasite relationship

Numerous observations of *I. multifiliis* infections in zebrafish larvae have provided us with theories on immune evasive behavior. One thing is clear – as long as the parasites are alive, they rotate and they rotate even faster when they are small and vulnerable (Dehai et al., 2000). Immune cells are most of the time prevented from interacting with the rotating parasite. Phagocytes struggled to get inside the interstitial space created by the parasite and neutrophils responded with NETs formation. From our timelapse observations, it appears that phagocytes move around the parasite, but their movement is much slower than the rotation of the early trophonts, hindering successful attachment and attack. Previously, it was believed that the rotation was a feeding strategy and a physical mechanism to keep an open space around the parasite (Dickerson and Dawe, 1995; Dehai et al., 2000). We propose that the rotation strategy of the parasite also is a major immune evasive strategy. It has been described how myxozoan parasites use a similar strategy to avoid host responses (Hartigan et al., 2016). Antibodies can bind to cilia and may stop the rotation, explaining why adaptive immunity with a high production of specific antibodies is efficient against these parasites (Clark et al., 1996; Jorgensen et al., 2011; Xu et al., 2013; Xu et al., 2016). When immune cells or other factors such as immunoglobulins manage to kill the parasite, the rotation stops (obviously) (**Supplementary Video 1**). We have only observed killing of parasites within the first 5-7 h of infection before the early trophont starts eating. When the mouth of the trophont is developed and it is capable of feeding, it can ingest and neutralize immune cells (Ventura and Paperna, 1985; Olsen et al., 2011; Jorgensen, 2016b). This has led us to believe that a severe battle between *I. multifiliis* and the zebrafish host, determining how severe the infection will become, lies in the early phase of infection before the early trophonts transform into feeding trophonts. It has been described that zebrafish appear more resistant towards the parasite than other species of fish (Jorgensen, 2016a), and here we document that phagocytes play a role in natural protection in zebrafish.

Single parasites create an interstitial space and secrete proteases and proteins involved in proteolytic and phagocytic activities during invasion, growth and development to destroy host tissue and immunosuppress the host locally (Jousson et al., 2007; Saleh et al., 2021). It has also been shown that the immunogenic GPI-anchored immobilization antigens originating from the parasite cilia and cell membrane are found in the surroundings of the parasite (Dickerson and Findly, 2014). This could act as a possible decoy mechanism where the host immune response is lured away from the real danger. Combined with its rotation, the parasite appears relatively safe in the interstitial space establishing host-parasite compatibility. If the early trophont leaves the interstitial space, a more severe response is triggered in the host tissue and the parasite becomes vulnerable to the host immune system, as described in the work by Ventura and Paperna in 1985 (Ventura and Paperna, 1985). It appears as if both the parasite and the host try to limit the reaction during mild infections, which would be the most beneficial situation in the wild. Heavier infections increase the immune responses and the subsequent protection level, indicating that the host responds according to the overall danger the parasite represents. Heavy infections, experienced in enclosed fish production systems, will cause too much damage to the susceptible hosts. The fish get exhausted and succumb to the disease and, at the same time, the parasite will not be able to continue its life cycle and dies. In this situation, the parasite and host are incompatible.

The innate immune response of the host is similar between mechanical injury and *I. multifiliis* infection in the early phase (Gonzalez et al., 2007a; Gonzalez et al., 2007b), indicating that the response is not related to the parasite, but mainly to the mechanical damage (DAMPs). It could, therefore, be suggested that the parasite must quickly create an interstitial space to protect itself from the more specific immune response. When the parasite grows, it ingests host immune cells (Ventura and Paperna, 1985; Olsen et al., 2011; Jorgensen, 2016b) and the host immune response becomes alleviated. These more mature parasites can move around unharmed under the host’s cell layers covering it.

To further document the role of neutrophils and macrophages in the response against *I. multifiliis* future studies should include the utilization of depletion lines (Rosowski, 2020). The parasites may be sensitive to the compound metronidazole, which is one way to initiate depletion, but other depletion methods should be applied as well. Studies of the parasite virulence factors should also be investigated to better understand the host-parasite interaction.

## Conclusion

White spot disease is a major problem for freshwater aquaculture all over the world and in this study, the immune response of the fish host and the parasite behavior were scrutinized. We can confirm that zebrafish larvae infected with *I. multifiliis* represent an excellent model to elucidate host-parasite relationships. Older larvae (12 dpf) were more resistant than younger larvae (5 dpf) indicating that older larvae manage to create a more hostile environment for the parasite. It was shown that phagocytes were often attracted to the parasite and tried to eliminate it. In some cases, immune cells successfully killed the parasite, notably with neutrophils utilizing NETs. The larvae reacted with mild, whole-body inflammation but in local microenvironments the phagocyte responses were dominating and severe. The parasite appeared to locate itself in an interstitial space and rotate vigorously in the early phase of infection, proposedly, as an immune evasive strategy to hide and protect itself from attacks by the phagocytes and possibly other factors of the host’s immune response.

## Data availability statement

The original contributions presented in the study are included in the article/**Supplementary Material**. Further inquiries can be directed to the corresponding author.

## Ethics statement

The animal study was reviewed and approved by The Animals Experiment Inspectorate under the Danish Ministry of Environment and Food with the license number 2021-15-0201-00951.

## Author contributions

HM, LJ and MM contributed to conception and design of the study. CG, CH, MM, JR, SG performed the experiments, sampling and data generation. SK-N captured and processed the images and videos. HM, LJ and MM performed the statistical analysis. LJ wrote the first draft of the manuscript. All authors contributed to manuscript revision, read, and approved the submitted version.

## Supplementary material

Supplementary Table 1 Gene assays used in the qPCR analyses. Sequence of primers, probes, length of product and GenBank accession number is shown.

Supplementary Video 1
*I. multifiliis* trophont surrounded by neutrophils (green, top left panel) and macrophages (red, top right panel). A dead neutrophil and subsequent green coloration in the interstitial space (arrows 1 and 2 respectively) and NETs formation were observed (arrow 3). The parasite can be observed in bright field (bottom left), the bottom right panel shows an overlay. The video shows a timelapse recording over 20 min, at the end of which the parasite dies (arrow 4).

Supplementary Video 2 Live imaging of the head of a five-day old zebrafish five hours after infection with *I. multifiliis*. A rotating parasite is found on the ventral side of the fish.

Supplementary Video 3 The part towards the tail fin after the anal opening of a twelveday old zebrafish five hours after infection with *I. multifiliis*. Three rotating parasites are found on the ventral side of the fish.

Supplementary Figure 1
**(A)** Four 6-well plates with 10 zebrafish larvae in each of all wells. Each well was infected with a certain concentration of the parasite *I. multifiliis* from 10 to 1280 theronts/larva. Twenty-four hours after infection the fish were anaesthetized, and the parasites were counted. This setup was used both for 5 and 12 dpf larvae. t = theronts. **(B)** Four 6-well plates with 10 zebrafish larvae in each of 18 wells. Half of the wells contained uninfected larvae as time point controls; the other half contained larvae infected with 50 *I. multifiliis* theronts/larva. Sampling was conducted at 2, 5 and 8 h after infection. This setup was used both for 5 and 12 dpf larvae. Created with Biorender.com.

Supplementary Figure 2 Phagocyte counting method. Cells were counted manually. **(A)** The whole fish with a square indicating the region of interest. **(B, C)** Counted neutrophils and macrophages, respectively, marked with a plus and a number. **(E, F)** Neutrophils and macrophages, respectively, in the region of interest. **F)** Region of interest illuminated with white light. **(G)** A merged image of **(D–F)**.

Supplementary Figure 3 An overview image of the location of the parasite (arrow) in **Supplementary Video 1** in a double transgenic 5 dpf zebrafish larvae with green fluorescent neutrophils and red fluorescent macrophages.

Supplementary Figure 4 Neutrophils and macrophages were counted in the region between the gat and the caudal fin in 5 and 12 dpf zebrafish larvae, respectively. There was no significant difference between the two age groups.
