# Supplementary figures and images for: Acute immune responses in zebrafish and evasive behavior of a parasite – who is winning?
Source: Front Cell Infect Microbiol. 2023 Jul 5;13:1190931. doi: 10.3389/fcimb.2023.1190931 (PMC10354369; doi:10.3389/fcimb.2023.1190931)

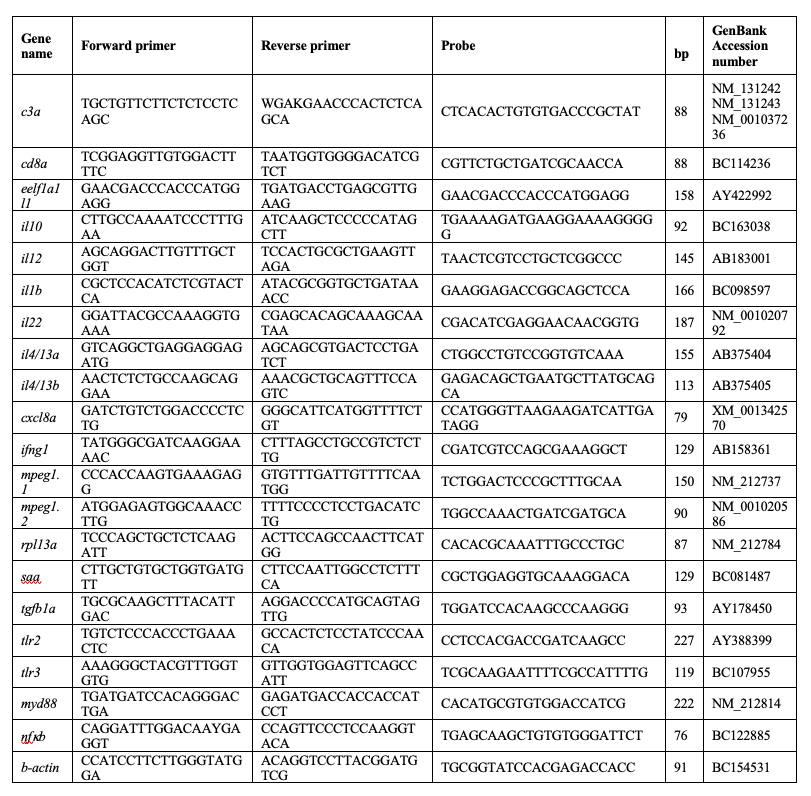

Supplement: Supplementary Table 1 — Gene assays used in the qPCR analyses. Sequence of primers, probes, length of product and GenBank accession number is shown. [file Table_1.docx]

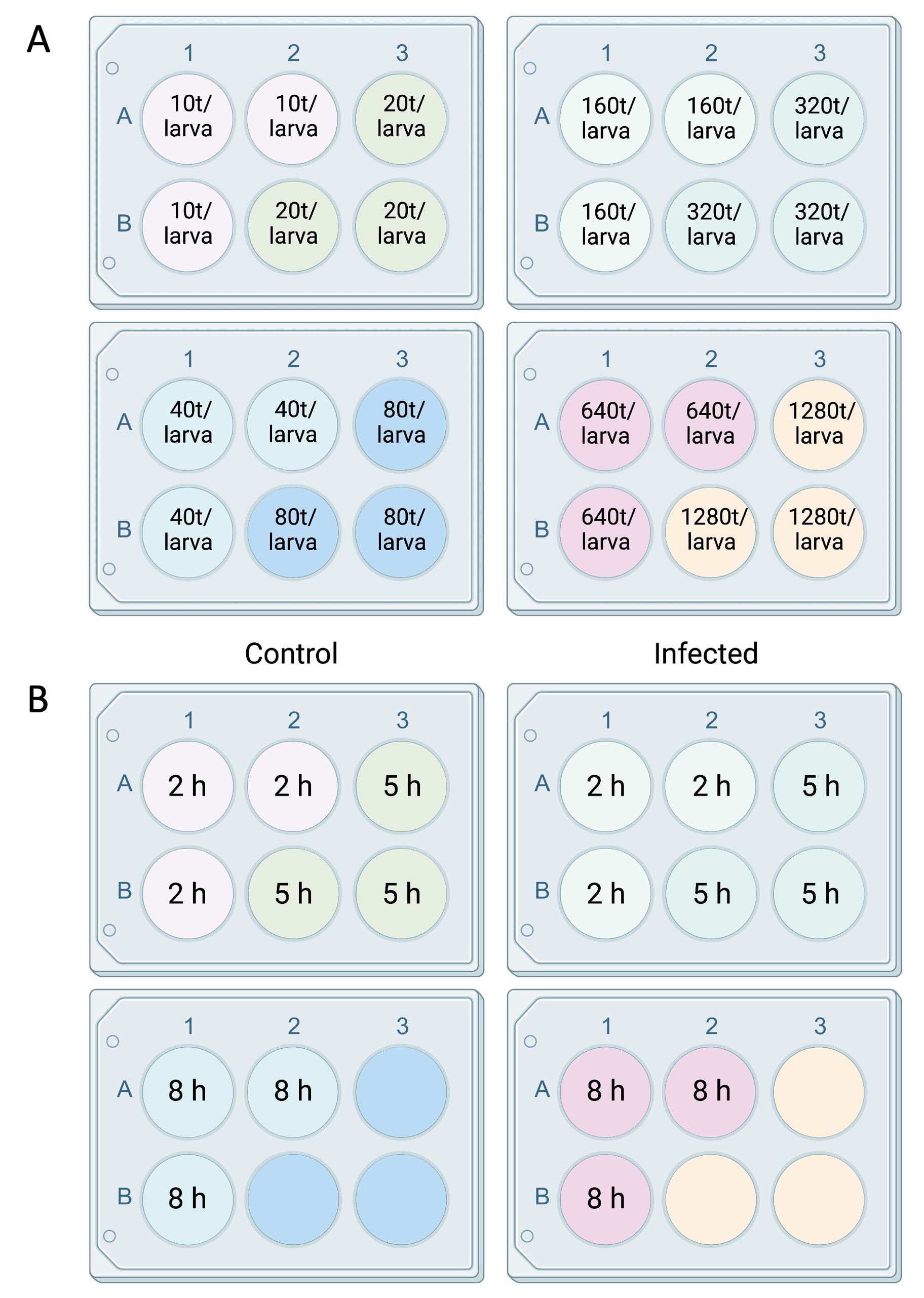

Supplement: Supplementary Figure 1 — (A) Four 6-well plates with 10 zebrafish larvae in each of all wells. Each well was infected with a certain concentration of the parasite I. multifiliis from 10 to 1280 theronts/larva. Twenty-four hours after infection the fish were anaesthetized, and the parasites were counted. This setup was used both for 5 and 12 dpf larvae. t = theronts. (B) Four 6-well plates with 10 zebrafish larvae in each of 18 wells. Half of the wells contained uninfected larvae as time point controls; the other half contained larvae infected with 50 I. multifiliis theronts/larva. Sampling was conducted at 2, 5 and 8 h after infection. This setup was used both for 5 and 12 dpf larvae. Created with Biorender.com. [file Image_1.jpeg]

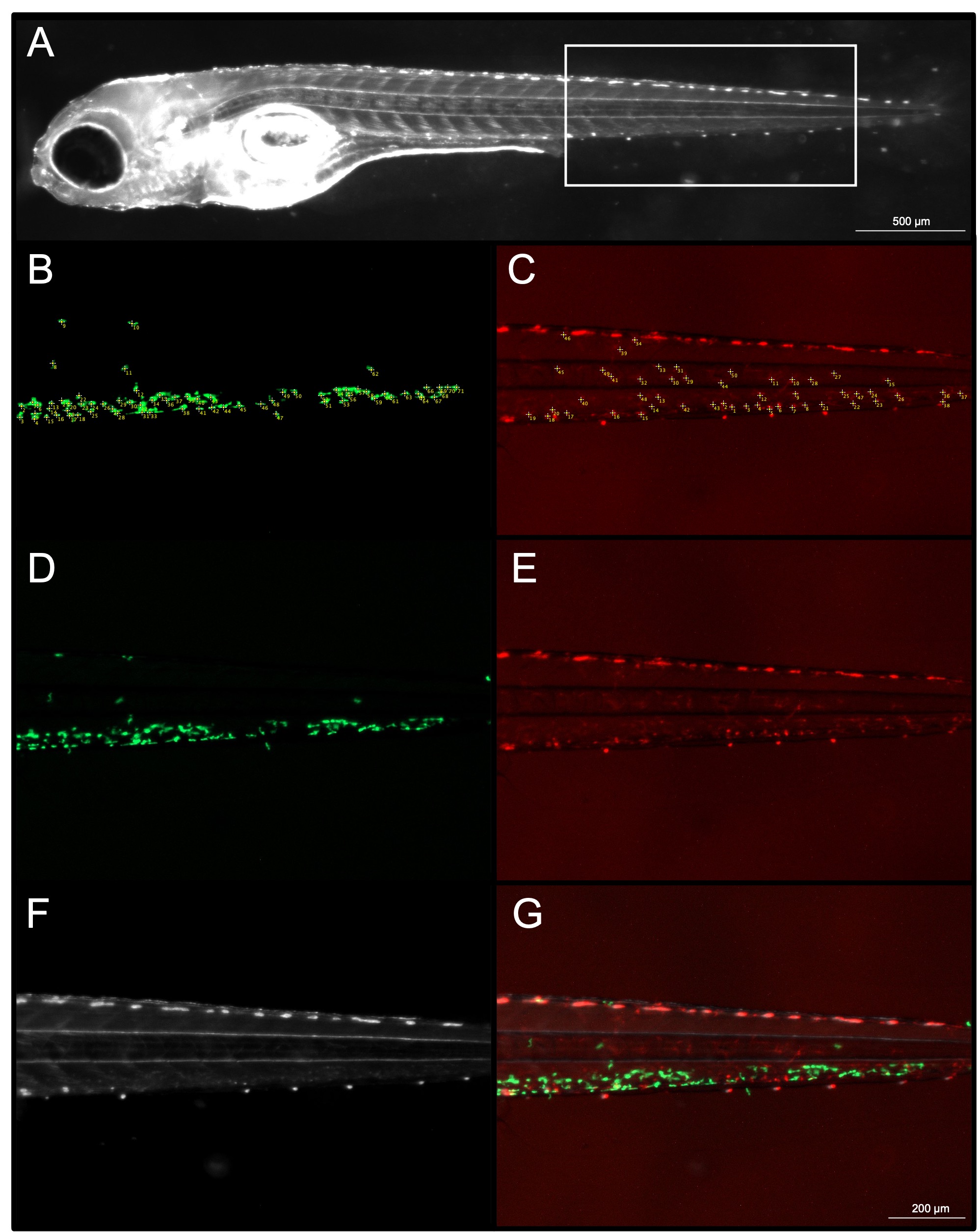

Supplement: Supplementary Figure 2 — Phagocyte counting method. Cells were counted manually. (A) The whole fish with a square indicating the region of interest. (B, C) Counted neutrophils and macrophages, respectively, marked with a plus and a number. (E, F) Neutrophils and macrophages, respectively, in the region of interest. F) Region of interest illuminated with white light. (G) A merged image of (D–F). [file Image_2.jpeg]

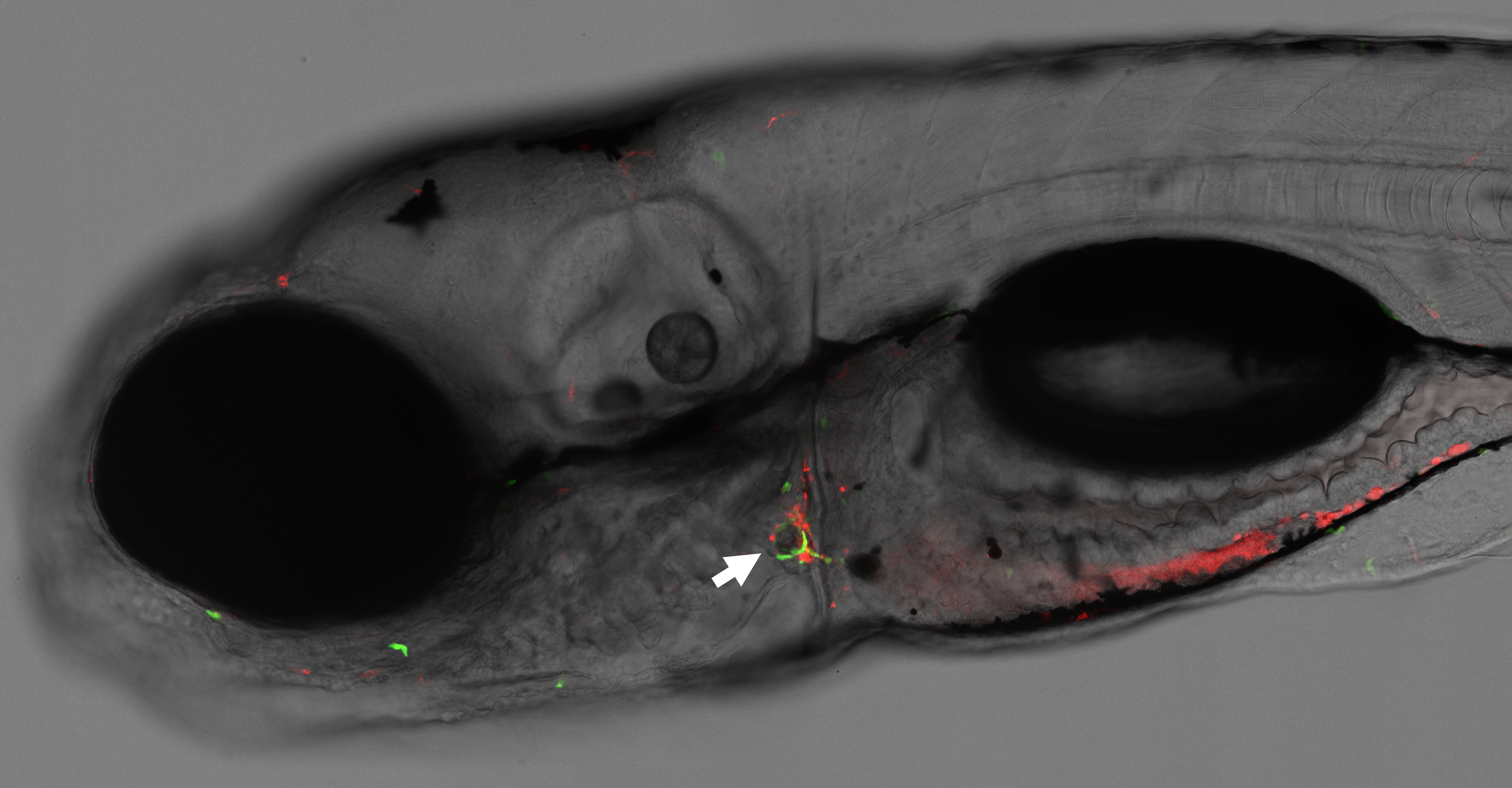

Supplement: Supplementary Figure 3 — An overview image of the location of the parasite (arrow) in Supplementary Video 1 in a double transgenic 5 dpf zebrafish larvae with green fluorescent neutrophils and red fluorescent macrophages. [file Image_3.jpeg]

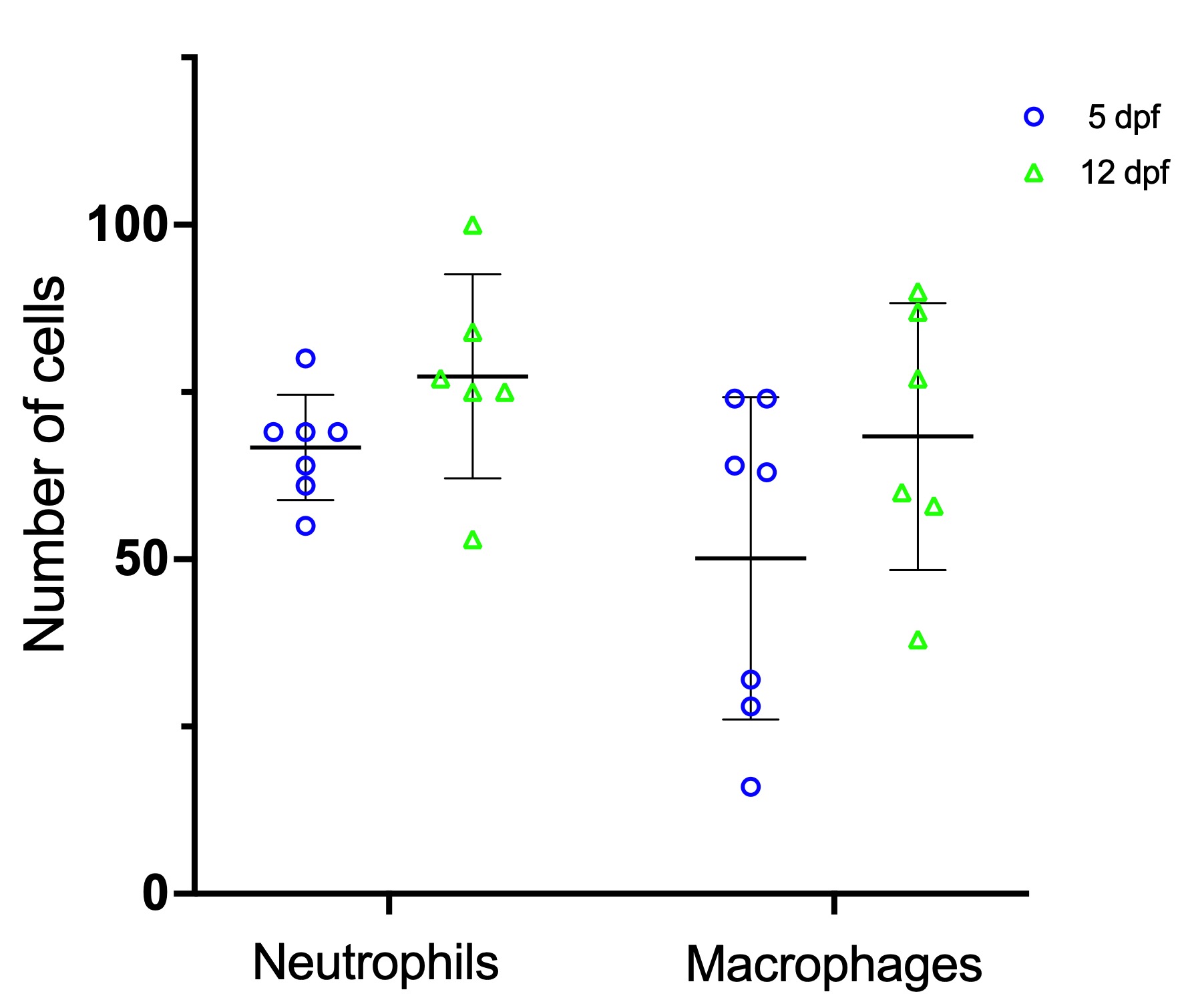

Supplement: Supplementary Figure 4 — Neutrophils and macrophages were counted in the region between the gat and the caudal fin in 5 and 12 dpf zebrafish larvae, respectively. There was no significant difference between the two age groups. [file Image_4.jpeg]
